# TUFT1 Promotes Triple Negative Breast Cancer Metastasis, Stemness, and Chemoresistance by Up-Regulating the Rac1/β-Catenin Pathway

**DOI:** 10.3389/fonc.2019.00617

**Published:** 2019-07-09

**Authors:** Weiguang Liu, Guanglei Chen, Lisha Sun, Yue Zhang, Jianjun Han, Yuna Dai, Jianchao He, Sufang Shi, Bo Chen

**Affiliations:** ^1^Department of Breast Surgery, Affiliated Hospital of Hebei University of Engineering, Handan, China; ^2^Department of Breast Surgery, Shengjing Hospital of China Medical University, Shenyang, China; ^3^Department of Physiology, Dalian Medical University, Dalian, China; ^4^Department of Breast Surgery, The First Hospital of China Medical University, Shenyang, China

**Keywords:** triple negative breast cancer (TNBC), TUFT1, Rac1, metastasis, stemness, chemoresistance

## Abstract

**Objectives:** Triple negative breast cancer (TNBC) is a subtype of breast cancer with stronger invasion and metastasis, but its specific mechanism of action is still unclear. Tuft1 plays an important regulatory role in the survival of breast cancer cells; however, its role in regulating TNBC metastatic potential has not been well-characterized. Our aim was therefore to systematically study the mechanism of TUFT1 in the metastasis, stemness, and chemoresistance of TNBC and provide new predictors and targets for BC treatment.

**Methods:** We used western blotting and IHC to measure TUFT1and Rac1-GTP expression levels in both human BC samples and cell lines. A combination of shRNA, migration/invasion assays, sphere formation assay, apoptosis assays, nude mouse xenograft tumor model, and GTP activity assays was used for further mechanistic studies.

**Results:** We demonstrated that silencing TUFT1 in TNBC cells significantly inhibited cell metastasis and stemness *in vitro*. A nude mouse xenograft tumor model revealed that TUFT1 knockdown greatly decreased spontaneous lung metastasis of TNBC tumors. Mechanism studies showed that TUFT1 promoted tumor cell metastasis and stemness by up-regulating the Rac1/β-catenin pathway. Moreover, mechanistic studies indicated that the lack of TUFT1 expression in TNBC cells conferred more sensitive to chemotherapy and increased cell apoptosis via down-regulating the Rac1/β-catenin signaling pathway. Further, TUFT1 expression positively correlated with Rac1-GTP in TNBC samples, and co-expression of TUFT1 and Rac1-GTP predicted poor prognosis in TNBC patients who treated with chemotherapy.

**Conclusion:** Our findings suggest that TUFT1/Rac1/β-catenin pathway may provide a potential target for more effective treatment of TNBC.

## Introduction

Triple negative breast cancer (TNBC) is a subtype of BC that lacks estrogen or progesterone receptors and has no epidermal growth factor receptor 2 amplification, accounting for about 20% of the total breast cancer (1–3). TNBC is defined mainly based on its pathology. Its features overlap with those of basal-like BC, one of five subgroups based on microarray gene expression profiling (4, 5). TNBC usually presents with less favorable clinical features than other subtypes of breast cancer, for example, tumors proliferate faster, relapse earlier and metastasis more easily and is usually associated with poorer prognosis as a result (6–8). However, the mechanism by which TNBC's metastasis is less clear. In addition, there are currently no very effective targeted drugs available for TNBC, cytotoxic chemotherapy remains the main adjuvant therapy for this subtype of breast cancer (9). A more in-depth study of the mechanism of TNBC metastasis may be able to more efficiently find its target, and at the same time provide theoretical support for the exploration of new TNBC therapeutic drugs.

Tuftelin (TUFT1) is an acidic, hydrophilic, glycosylated, and phosphorylated protein. Sequence and characterization analysis has shown that TUFT1 is well conserved, with high homology across various species. The protein is considered to act on enamel mineralization and is involved in the interaction between mesenchymal ectoderm and autosomal enamel dysplasia during tooth development (10). Zhou et al. (11) demonstrated that the expression of TUFT1 protein in pancreatic cancer is higher than that in normal pancreatic tissue. Its expression is closely related to both the disease stage and local lymph node metastasis. Cell function experiments further confirmed that TUFT1 depletion reduced proliferation and metastasis of pancreatic cancer cells, and impaired various proteins expression related to epithelial-mesenchymal transition. The authors suggested that TUFT1 may affect HIF1 by influencing the expression of members of the Snail signaling pathway, which regulates epithelial mesenchymal transition. Our previous study found that inhibition of TUFT1 expression in breast cancer cells inhibited proliferation, affected the cell cycle, and induced apoptosis. In addition, we showed that suppression of TUFT1 affected the expression of the proteins RelA, Caspase 3, DUSP1, and Rac1 (12, 13). Kawasak et al. (14) found that TUFT1 activated the mTORC1 signaling pathway by regulating the Rab GTPase, and that the interaction between TUFT1 and RabGAP1 mediated intracellular lysosome localization and vesicle transport in tumor cells. However, the precise role of TUFT1 in breast cancer (BC), including the mechanics of TNBC's metastasis remain unclear.

Rac1 is a member of the Rho GTPases family, which is a subgroup of the Ras superfamily (15). Rac1 is activated by binding to GTP, while it is deactivated by binding to GDP, which makes it play an important role in many signaling pathways (16). Rac1 plays an important role in cancer progression (17), affecting cell adhesion, proliferation, migration, invasion, and cancer metastasis (18–20). The new study highlights the importance of Rac1 activation in cancer metastasis and acquired chemoresistance (21–24). One major mechanism by which Rac1 may provide resistance to chemotherapy is its role in apoptosis regulation. Rac1-GTP can bind directly to the key apoptotic regulator Bcl-2 to elicit anti-apoptotic cell responses (25). Many studies have also proved that Rac1-GTP can affect the genes Nanog, Sox2, and Oct4, which play a central regulatory role in CSC (26–28). Rao et al. (29) showed that Rac1/β-catenin pathway participated in SEMA3F-mediated regulation of colorectal cancer cell stemness. In addition, Kawasak et al. (14) found that TUFT1 increased Rac1 levels through activation of the AKT/mTOR pathway. However, the functional mechanism of TUFT1 in metastasis, stemness, and chemoresistance of BC, especially in TNBC, has not been adequately characterized.

In this study, we showed that stable TUFT1 knockdown in TNBC cells drastically inhibited their migration, invasiveness, and CSC-like properties. Moreover, we found that the expression of TUFT1 increased significantly in TNBC samples. The co-expression of TUFT1 and Rac1-GTP suggested poor prognosis. Further functional studies showed that TUFT1 promoted TNBC cell metastasis, stemness, and chemoresistance by up-regulating the Rac1/β-catenin signaling pathway.

## Materials and Methods

### Human Specimens

In our study, we recruited 60 pathologically confirmed TNBC patients at Affiliated Hospital of Hebei University of Engineering, between January 2014 and December 2014. All patients treated with anthracycline followed by taxanes chemotherapy after surgery. This study was carried out in accordance with the recommendations of ICMJE with written informed consent from all subjects. All subjects gave written informed consent in accordance with the Declaration of Helsinki. The protocol was approved by the Ethics Committee of Affiliated Hospital of Hebei Engineering University.

### Human BC Cell Lines and Plasmids

HCC1937 cell line was obtained from the American Type Culture Collection (USA). MDA-MB-231 cell line was gained from the Chinese Academy of Sciences (China). Cells were cultured in RPMI-1,640 mixed with 10% FCS in an atmosphere containing 5% CO_2_. Recombinant retroviruses carrying PLNCX2-vector or PLNCX2-TUFT1 were synthesized based on relevant instructions (Clontech). MDA-MB-231 or HCC1937 cells with Polybrene [8 μg/mL (Sigma-Aldrich)] were infected these retroviruses and then were selectively isolated with G418 [750 μg/mL (Calbiochem)].

### RNA Interference

The recombinant adenoviruses encoding 2 different short-hairpin RNAs (shRNAs), respectively specific for human TUFT1 were designed and prepared from company (GeneChem, Shanghai, China).TUFT1-shRNA#1: AGAGAATTTAGAGATGCAT; TUFT1-shRNA#2: GGTGGAGTATTTACGGTAAAC. Lentiviruses were transfected into cells based on the relevant instructions. The ability of TUFT1 knockdown was assessed by real-time quantitative PCR and western bolt. Cell lines with over 80% efficacy were considered stable. More than 80% of the cell transfection efficiency was considered stable.

### IHC Analyses

TUFT1 (dilution 1:100, Abcam, USA), RAC1-GTP (dilution 1:800, NewEast Bioscience, USA), were purchased. The experimental method was carried out and the expression of TUFT1 and RAC1-GTP was evaluated semi-quantitatively according to the criteria described previously (12, 13). The analysis was performed by two independent pathologists.

### Real-Time PCR

Total RNA was extracted by Trizol (Invitrogen) for reverse transcription, according to manufacturer's instructions (Invitrogen). TUFT1 expression was examined by Real-time PCR according to the criteria described previously (12, 13).

### Western Blot

The rabbit antibodies used to detect TUFT1, Rac1, β-catenin, Nanog, SOX2, and OCT4 were obtained from AbCam (Cambridge, UK). Their protein levels were examined by western blot according to the criteria described previously (12, 13).

### Wound Healing Assay

Following the manufacturer's recommendations, marker pen was used on the back of the 6-well plate, horizontal lines were evenly drawn, about 2 × 10^5^ cells were added, and the next day, the gun head was scratched. Cells were washed with PBS for 3 times and serum-free medium was added. Incubate in a 37°C, 5% CO2 incubator. Sample at 0, 8, 24 h and take photos.

### Invasion Assay

The required number of chambers were placed in a new 24-well plate and 500 μL serum-free medium was added to the upper and lower chambers, respectively. The preparation of serum-free cell suspension is usually 5 × 10^4^ cells/well (24-well plate). 500 μL cell suspension was added to the upper chamber and 750 μL 30% FBS medium was added to the lower chamber. The incubator was incubated at 37°C for 24 h. Hematoxylin and eosin (H&E) stained cells to the lower surface of the membrane. Photographs are taken under a microscope.

### Transwell Assay

Serum-free cell suspension was prepared and counted, usually 5 × 10^4^ cells/well (24-well plate). Carefully remove the culture medium in the upper chamber and add 100 μL cell suspension. Add 600 μL 30% FBS culture medium in the lower chamber. The incubator was incubated at 37°C for 24 h. The chamber was fixed in 4% paraformaldehyde for half an hour. 1–2 drops of staining solution were used to stain and transfer cells to the lower surface of the membrane for 1–3 min. Photographs are taken under a microscope.

### Sphere Formation Assay

Cell trypsin of each experimental group in the logarithmic growth phase was digested, serum-free medium was resuspended, cell suspensions were made, and counted. The cell suspension was inoculated in the ultra-low adhesion 6-well plate culture plate at a density of 10,000–20,000 cells/wells, and 2 mL serum-free medium DMEM/F12 was added to each well. Will the good cells in under the condition of 37°C and 5% CO2, every 2–3 days in liquid, extend the every 6–8 days. Observe cell balling and morphology under microscope at any time.

### Apoptosis Assay

After infection, supernatant was collected from cell culture in each experimental group in a 5 ml centrifuge tube. The cells were washed once by D-Hanks, the cells were digested by trypsin, and the culture supernatant was terminated. The cells were collected in the same 5 ml centrifuge tube. Centrifuge 1,500 rpm for 5 min and discard the supernatant. The cells were washed with PBS and precipitated once, centrifuged at 1,500 rpm for 5 min, and the cells were collected. The cells were washed with 1 × binding buffer for once, centrifuged at 1,500 rpm for 5 min, and the cells were collected. Cell suspension of 100 μl (1 × 10^5^-1 × 10^6^ cells) was taken and stained with PI complex dyeing liquor (0.5 mL) for 10–15min at room temperature. Flow cytometry was used for detection.

### Rac1–GTP Pull-Down Assay

Cells were splitting in buffer including 25 mM HEPES, 1% NP40, 10% glycerin, 5 mM MgCl2, 1 mM DTT, 100 mM NaCl, and protease inhibitors. The pyrolysate was cultured on ice for 5 min and centrifuged for 1 min with 10,000 × g. Post-nuclear supernatant was tested for pull-down analysis of 30 μg GST-RBD (Rac1) pre-coated GSH beads in each case. The beads and supernatant were cultured in a table at 4°C for 15 min. The beads were washed with a solution buffer containing 0.01% NP40, boiled with SDS PAGE, and separated. The beads were analyzed by Western blotting as shown above. NSC23766 (obtained from Tocris Bioscience) was used to inhibit Rac1 activation.

### Tumor Metastasis and Growth in Nude Mice

4–6-weeks female nude mice were obstained from the Shanghai Lingchang Biological Technology Ltd (Shanghai, China). The caudal vein was selectively injected into ShTUFT1—MDA-MB-231 cells. The nude mice were anesthetized by isoflurane gas using *in vivo* imaging instrument with gas anesthesia system. The mice were sacrificed at 10 weeks after treatment, and metastatic lung nodules were counted.

For the *in vivo* chemoresistance experiment, shTUFT1—MDA-MB-231 cells were injected into the flanks of nude mice (10 mice/group). Each group was divide randomly into two subgroups after 2 weeks that were either left untreated or received intraperitoneal injections of doxorubicin (4 mg kg^−1^) every 5 days(three cycles), as previously described by Ghebeh et al. (30). Animal handling and research protocols were approved by the Ethics Committee of Affiliated Hospital of Hebei Engineering University.

### ONCOMINE Analysis

The mRNA levels of TUFT1 in BCs were determined through analysis of data from the *ONCOMINE* database (www.oncomine.org). In our study, BC specimen data were compared with control datasets using student's *t*-test to examined the *p*-value. The fold change was defined as 2, and the *p*-value was set up at 0.01.

### Statistical Analysis

The SPSS 23.0 software was used for statistical analyses. Student's *t*-test and Pearson correlation test were used to compare the classified variables. *p* < 0.05 was considered significant.

## Results

### TUFT1 Regulates Metastasis and Stemness of TNBC Cells *in vitro* and *in vivo*

**Figure 1:**
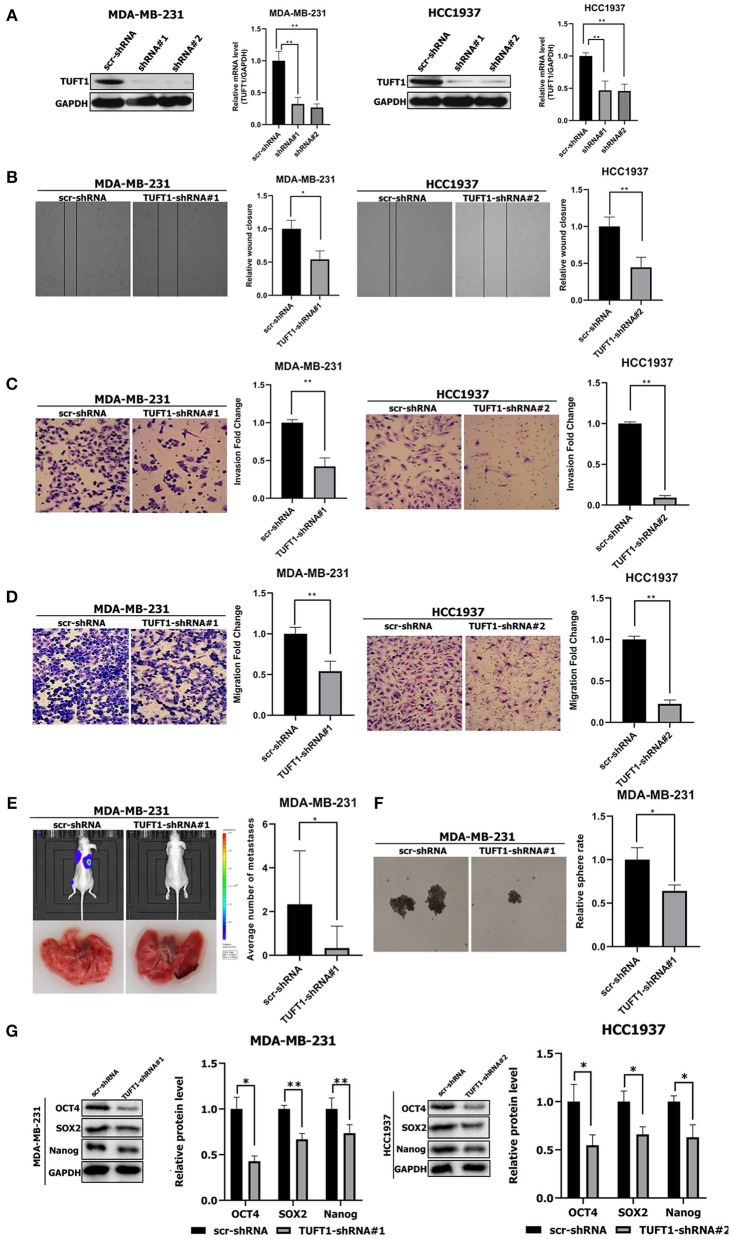
Effects of TUFT1 on migration, invasiveness and stemness *in vivo* and *in vitro*. MDA-MB-231 and HCC1937 cells were infected with TUFT1-shRNA or scramble (scr)-shRNA. **(A)** TUFT1 protein and mRNA expression levels were reduced in MDA-MB-231 and HCC1937 cells with infection of adenovirus encoding TUFT1-shRNA#1 and TUFT1-shRNA#2. Cell wound **(B)**, invasiveness **(C)** and migration **(D)** were aberrant regulated after TUFT1 down-regulation in MDA-MB-231 or HCC1937 cells (*n* = 3). **(E)** TUFT1 knockdown in MDA-MB-231 cells significantly reduced the number of lung metastatic nodules (*n* = 10). **(F)** TUFT1 knockdown drastically reduced the number of mammary spheres formed by MDA-MB-231 cells (*n* = 3). **(G)** The protein levels of Nanog, Sox2 and Oct4 were decreased by TUFT1 knockdown in both MDA-MB-231 and HCC1937 cells (*n* = 3). Results are presented as means ± SD. The statistical significance was assessed by student's *t*-test; ^*^p < 0.05, ^**^p < 0.01.

First, we performed TUFT1 knockdown in the HCC1937 and MDA-MB-231 TNBC cell lines using shRNA. Western blot and Real-time PCR revealed that TUFT1 protein and mRNA levels were prominently reduced in TUFT1-knockdown cells compared to control cells (*p* < 0.01, Figure 1A). Wound healing assays, invasion assays, and transwell assays were all used to examined the role of TUFT1 on the migration of TNBC cells. We found that TUFT1 down-regulation markedly reduced the migration of both TNBC cells compared to control cells, indicating that TUFT1 knockdown inhibits cell migratory ability (*p* < 0.05, Figures 1B–D).

To expand on our study *in vitro*, we next examined if TUFT1 could promote the metastasis in TNBC cells. ShTUFT1- MDA-MB-231 cells were injected into the caudal vein of nude mice. Then mice were sacrificed for quantitative analysis of lung metastatic nodules. Mice injected with ShTUFT1- MDA-MB-231 cells developed significantly fewer metastatic lung nodules than control mice (*p* < 0.05, Figure 1E). Taken together, results *in vitro* and *in vivo* reveal the metastatic potential of TUFT1 in TNBC cells.

CSCs play a key role in cancer metastasis (31, 32). We used a sphere formation assay to examined the role of TUFT1 on the stemness of TNBC cells. We found that TUFT1 knockdown drastically reduced the number of mammary spheres formed by MDA-MB-231 cells (*p* < 0.05, Figure 1F). Nanog, Sox2, and Oct4 play a central regulatory role in CSCs (26–28, 33, 34). We found that the Nanog, Sox2 and Oct4 levels were reduced by TUFT1 knockdown in both MDA-MB-231 and HCC1937 cells (*p* < 0.05, Figure 1G). These results reveal that TUFT1 is capable of significantly promoting CSC-like properties in TNBC cells.

### TUFT1 Promotes the Metastasis of TNBC Cells by Up-Regulating the Rac1/β-Catenin Pathway

**Figure 2:**
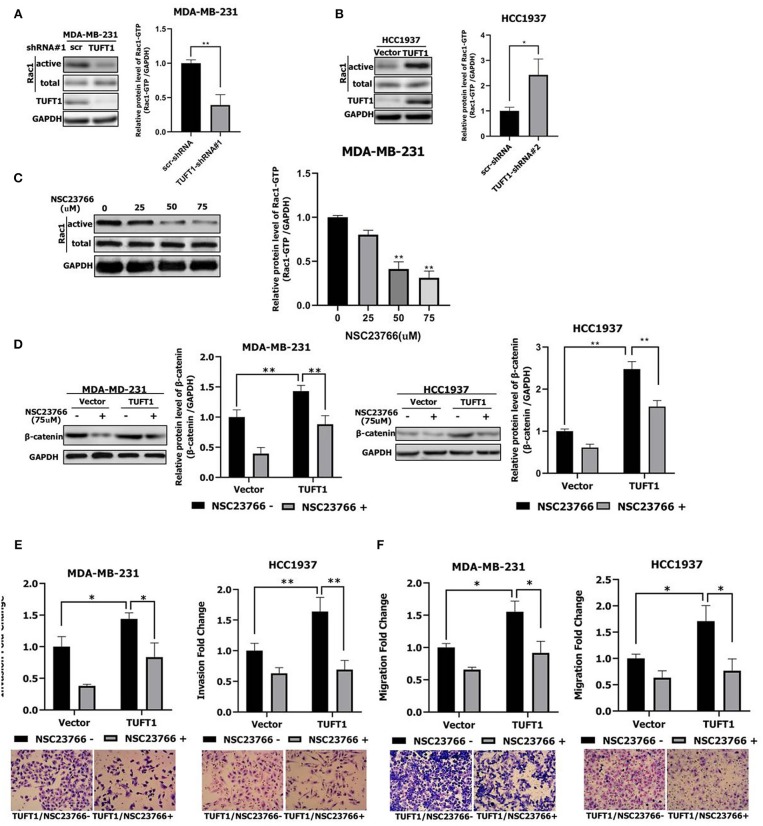
Role of TUFT1 in TNBC migration and invasiveness by promoting the Rac1/β-catenin signaling pathway. **(A)** Down-regulation of TUFT1 by shRNA dramatically decreased the protein level of Rac1-GTP in MDA-MB-231 cells (*n* = 3). **(B)** Overexpression of TUFT1 increased the protein level of Rac1-GTP in HCC1937 cells (*n* = 3). **(C)** Western blot showed effect of Rac1 inhibitor NSC23766 on Rac1-GTP in MDA-MB-231 cells (*n* = 3). **(D)** Down-regulation of Rac1-GTP treated with NSC23766 decreased expression of β-catenin in TUFT1—MDA-MB-231 and TUFT1—HCC1937 cells (*n* = 3). Down-regulation of Rac1-GTP treated with NSC23766 decreased cell invasiveness **(E)** and migration in **(F)** in TUFT1—MDA-MB-231 and TUFT1—HCC1937 cells (*n* = 3). Results are presented as means ± SD. The statistical significance was assessed by student's *t*-test; ^*^p < 0.05, ^**^p < 0.01.

To further investigate TUFT1-regulated metastasis in TNBC cells, we performed Rac1 activity assays following manipulation of TUFT1 expression levels. This revealed that knockdown of endogenous TUFT1 decreased Rac1–GTP levels in MDA-MB-231 cells (*p* < 0.01, Figure 2A), whereas TUFT1 overexpression increased Rac1–GTP levels in HCC1937 cells (*p* < 0.05, Figure 2B). These data indicate that TUFT1 promotes Rac1 activation in TNBC cells.

To investigate the potential role of Rac1 downstream of TUFT1, endogenous Rac1-GTP was inhibited using the Rac1 inhibitor NSC23766 (29) in TUFT1 overexpression TNBC cells. We confirmed that NSC23766-mediated inhibition of Rac1 was associated with a substantial reduction in its active form, Rac1–GTP (Figure 2C). The activation of Wnt/β-catenin pathway is related to the proliferation and metastasis of TNBC (35, 36). Interestingly, we found that β-catenin levels were significantly increased by TUFT1 overexpression in both TNBC cells (*p* < 0.01, Figure 2D). However, the increase in β-catenin induced by TUFT1 overexpression was significantly decreased by NSC23766 treatment in both TNBC cells, compared to the controls (*p* < 0.01, Figure 2D). Consistent with this, we observed that TUFT1-dependent TNBC cells metastasis was reversed in cells treated with NSC23766, as assessed by both invasion and transwell assays (*p* < 0.05, Figures 2E,F). In conclusion, these results suggest that Rac1 is necessary for TUFT1-dependent β-catenin activation and TNBC cells metastasis.

### TUFT1 Promotes the Stemness of TNBC Cells by Up-Regulating the Rac1 Signaling Pathway

**Figure 3:**
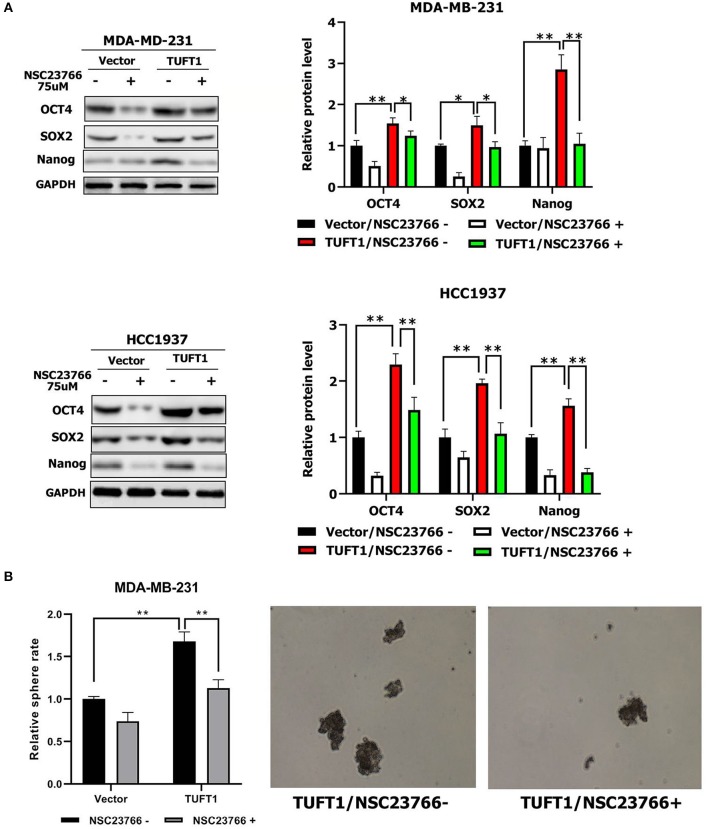
Role of TUFT1 in TNBC stemness by promoting the Rac1 signaling pathway. **(A)** Down-regulation of Rac1-GTP treated with NSC23766 decreased expression of Nanog, Sox2, and Oct4 levels in TUFT1—MDA-MB-231 and TUFT1—HCC1937 cells (*n* = 3). **(B)** Down-regulation of Rac1-GTP treated with NSC23766 decreased number of mammary spheres formed by TUFT1 - MDA-MB-231 cells (*n* = 3). Results are presented as means ± SD. The statistical significance was assessed by student's *t*-test; ^*^p < 0.05, ^**^p < 0.01.

To further investigate the regulation of TNBC cell stemness by TUFT1, we once again employed the Rac1 inhibitor NSC23766 (29) to inhibit endogenous Rac1-GTP in both TUFT1 overexpression TNBC cells. We found that Nanog, Sox2, and Oct4 levels were significantly increased by TUFT1 overexpression in both TNBC cells (*p* < 0.05, Figure 3A). However, the TUFT1-induced increase in Nanog, Sox2, and Oct4 was significantly decreased by NSC23766 treatment in both TNBC cells, compared to the corresponding controls (*p* < 0.05, Figure 3A). Consistent with this, we observed that NSC23766 treatment in MDA-MD-231 cells impaired TUFT1-dependent CSC-like properties, as assessed by the sphere formation assay (*p* < 0.01, Figure 3B).

### TUFT1 Inhibits Chemotherapy-Mediated Apoptosis in TNBC Cells by Targeting the Rac1/β-Catenin Signaling Pathway

**Figure 4:**
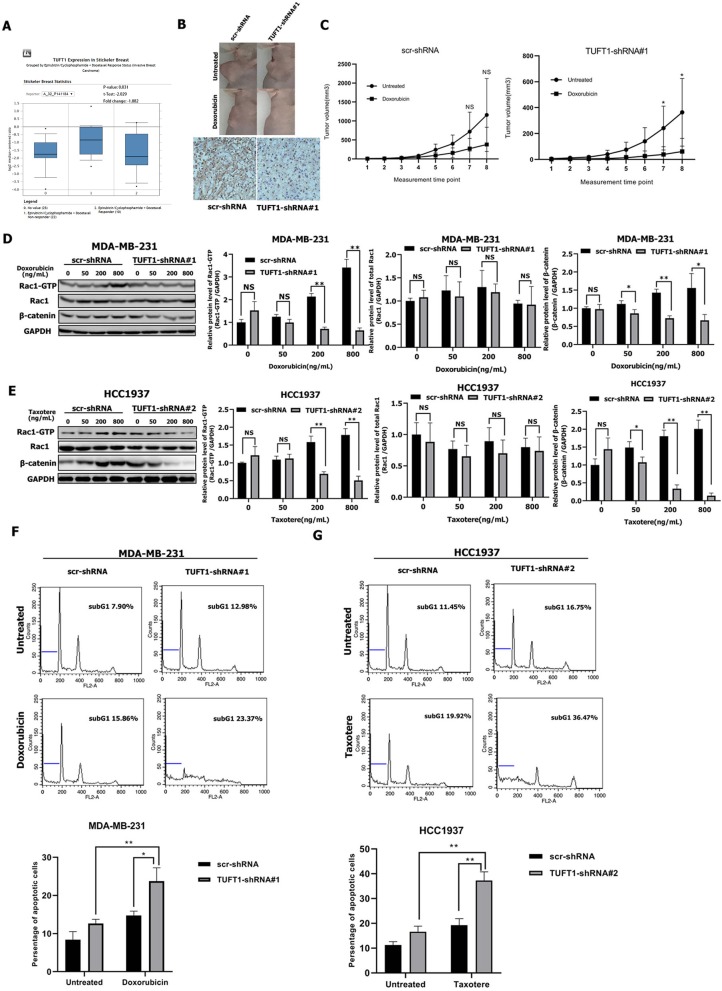
TUFT1-knockdown TNBC cells are more sensitive to doxorubicin and taxotere. **(A)** TUFT1 mRNA expression was lower in epirubicin/docetaxel responder BC samples by ONCOMINE analysis. **(B)** scr-shRNA- and TUFT1-shRNA-MDA-MB-231 cells were injected into nude mice as described in the Materials and Methods. The tumor volumes were measured following treatment with or without doxorubicin (*n* = 5). Representative images showing tumor formed in nude mice after injection with scr-shRNA- or TUFT1-shRNA cells and IHC staining of TUFT1 in tumor tissues. **(C)** Tumor volumes in four groups. **(D,E)** Western blot showing the expression levels of Rac1-GTP, Rac1 and β-catenin in scr-shRNA- and TUFT1-shRNA-MDA-MB-231 cells following treatment with various doses of doxorubicin or TUFT1-shRNA-HCC1937 cells following treatment with various doses of taxotere for 24 h (*n* = 3). **(F,G)** Apoptotic cell death was detected by PI single staining method following treatment of scr-shRNA- and TUFT1-shRNA-MDA-MB-231 cells without or with 200 ng ml^−1^ of doxorubicin or TUFT1-shRNA-HCC1937 cells without or with 200 ng ml^−1^ of taxotere for 24 h (*n* = 3). Numbers in the subG1 phase (blue bar) represent the percentage of apoptosis. Results are presented as means ± SD. The statistical significance was assessed by student's t-test; ^*^p < 0.05, ^**^p < 0.01.

*ONCOMINE* data showed that TUFT1 mRNA levels were significantly lower in epirubicin/docetaxel responder BC samples than epirubicin/docetaxel non-responder BC samples (*p* = 0.031, Figure 4A). To evaluate whether TUFT1 expression can directly contribute to resistance to chemotherapy in TNBC, we used MDA-MB-231-shTUFT1 cells (or control MDA-MB-231 cells) in a xenograft tumor model. IHC staining revealed that the tumors formed by the MDA-MB-231-TUFT1-shRNA cells had lower TUFT1 expression than those formed by the control cells (Figure 4B). The size of tumors formed by TUFT1-positive cells was slightly reduced by doxorubicin treatment (*p* > 0.05, Figure 4C), whereas the size of the tumors formed by TUFT1-negative cells was significantly reduced by doxorubicin treatment (*p* < 0.05, Figure 4C). These results show that the expression of TUFT1 is directly related to the increase of chemoresistance.

We next wondered whether TUFT1 confers resistance to chemotherapy in TNBC cells via the Rac1/β-catenin signaling pathway. Treatment of TUFT1-negative MDA-MB-231 cells with doxorubicin and HCC1937 cells with taxotere induced a decrease in both Rac1-GTP and β-catenin levels in a dose-dependent manner (Figures 4D,E). The protein levels of Rac1-GTP and β-catenin were significantly lower in TUFT1-negative cells than in TUFT1-positive cells following treatment with corresponding dose of doxorubicin and taxotere (*p* < 0.05, Figures 4D,E). However, the level of total Rac1 protein was unchanged (Figures 4D,E). Furthermore, we observed a significantly higher level of apoptosis in TUFT1-negative cells than in TUFT1-positive cells following treatment with 200 ng/mL doxorubicin or taxotere (*p* < 0.05, Figures 4F,G). These results indicate that TUFT1 may confer resistance to chemotherapy in TNBC cells by promoting cell apoptosis via the Rac1/β-catenin signaling pathway.

### TUFT1 and Rac1-GTP Expression Positively Correlate and Predict Poor Prognosis Following Treatment With Chemotherapy in TNBC

**Figure 5:**
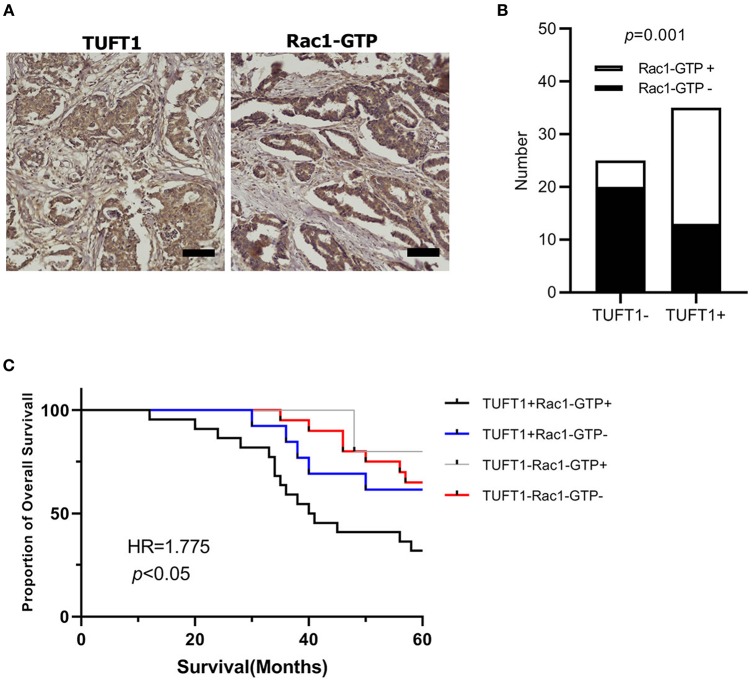
The expression of TUFT1 and Rac1-GTP in 60 TNBC patients who had received anthracycline/taxanes chemotherapy after surgery. **(A)** Show the positive expression of TUFT1 and Rac1-GTP in serial sections. **(B)** Rac1-GTP positively correlated with TUFT1 expression in the TNBC samples. **(C)** Kaplan–Meier survival curves showing survival in 60 patients who received chemotherapy blotted in relation to TUFT1 and Rac1-GTP expression. Survival curves showing the poor overall survival in patients with tumors co-expressing TUFT1 and Rac1-GTP that received chemotherapy.

We next studied the clinical correlation of TUFT1 and Rac1-GTP using 60 TNBC specimens from patients who had received anthracycline followed by taxanes chemotherapy after surgery. Examples of positive expression of TUFT1 and Rac1-GTP in serial sections are presented in Figure 5A. The level of TUFT1 protein was positively correlated with tumor size, histological grade and axillary lymph node metastasis (*p* = 0.010, *p* = 0.005, and *p* = 0.010, respectively, Table 1). The level of Rac1-GTP protein positively correlated with TUFT1 expression in the TNBC samples (*p* = 0.001, Table 2; Figure 5B). We divided the patients into four groups according to the TUFT1 and Rac1-GTP expression in the TNBC samples. Our patient follow-up analysis showed that a total of 27 of 60 patients died, and the 5-years overall survival rate was 55.0%. Fourteen of the 22 patients with tumors co-expressing TUFT1 and Rac1-GTP were dead, and this group displayed the lowest 5 years survival than other groups (log-rank test, *p* < 0.05, Hazard Ratio = 1.775, 95% CI of ratio = 0.986–3.195, Figure 5C). Therefore, TUFT1 and Rac1-GTP expression positively correlate and predict patient prognosis following treatment with chemotherapy in TNBC.

**Table 1:** The relationship between TUFT1 expression and the clinicopathological factors in TNBC patients who have received chemotherapy (*n* = 60).

**Variable**	***n***	**TUFT1^**−**^**	**TUFT1^**+**^**	***p*-variable**
Age				0.695
≥40	49	21	28	
<40	11	4	7	
Tumor size				0.010
T1	16	11	5	
T2–4	44	14	30	
Histological grades				0.005
I, II	28	17	11	
III	32	8	24	
Lymph node metastasis				0.010
−	18	12	6	
+	42	13	29	

“*+,” positive; “–,” negative*.

**Table 2:** Correlations between expression of TUFT1 and Rac1-GTP.

**Variable**	***n***	**Rac1-GTP^−^**	**Rac1-GTP^**+**^**	***p*-variable**
TUFT1				0.001
–	25	20	5	
+	35	13	22	

“*+,” positive; “–,” negative*.

## Discussion

To our knowledge, this is the first systematic study on the functional mechanism of TUFT1 mediated metastasis and stemness in TNBC. Zhou et al. (11) reported that TUFT1 overexpression promoted the metastasis of pancreatic cancer cells, and affected the expression of a number of epithelial-mesenchymal transformation-related proteins. They suggested that TUFT1 may affect HIF1 by influencing the expression of members of the Snail signaling pathway, which regulates epithelial-mesenchymal transition. Kawasak et al. (14) found that TUFT1 may be activated by the AKT/mTOR pathway to regulate tumor proliferation and metastasis. Compared to cells of other breast cancer subtypes, basal mesenchymal-like TNBC cells display increased migration, invasion, and metastatic potential (37). In this study, we found that TUFT1 promotes the metastasis of TNBC cells both *in vitro* and *in vivo*. CSCs have high tumorigenic capacity and are important features of new tumors (secondary and third foci) at locations other than those of the original tumor (38, 39). Here, we propose for the first time that TUFT1 can regulate the stemness of TNBC cells. TUFT1 knockdown in TNBC cells reduced the number of mammary spheres and stemness-associated molecules. These results reveal that TUFT1 may promote the metastasis of TNBC cells by up-regulating their stem capacity.

Rac1, a member of the Rac subfamily of small GTPases, has its forms of active GTP-bound and inactive GDP-bound. Rac1 activity plays roles in the regulation of proliferation, differentiation, apoptosis, cell movement, and adhesion. Moreover, Rac1 has been shown to have an important role in tumor cell migration (40). Rac1-GTP interacts with different downstream effector molecules, thus affecting tumor invasion and metastasis (41). β-catenin, a target molecule of Rac1, is a key regulator of cell proliferation and metastasis (42, 43). β-catenin is a multi-gene nuclear transcription target. It can regulate the proliferation and metastasis of cancer cells (44, 45). Rac1 gene regulates β-catenin and locates its nucleus at the promoter TCF3/4 of target gene (46). Furthermore, active/inactive Rac1 state was shown to direct Rac1-β-catenin complex to the nucleus in CRC cells (47). De et al. (36) demonstrated that Rac1 was activated by cascade of β-catenin-Tiam1/vav2 as downstream target of Wnt/β-catenin pathway activation during TNBC metastasis. However, our results show that TUFT1 can promote the metastasis of TNBC cells by activating Rac1 in the Rac1/β-catenin signaling pathway, suggesting that the TUFT1/Rac1/β-catenin axis may regulate metastasis in TNBC. NSC23766 reduces total β-catenin in CRC cells, thus demonstrating that Rac1 regulates stemness in CRC by activating Wnt/β-catenin signaling (29). Our study further implicates Rac1 and its downstream target β-catenin as critical molecules in the regulation of stemness in TNBC downstream of TUFT1. Our study identifies the TUFT1/Rac1/β-catenin axis as a novel regulator of metastasis and stemness in TNBC. However, how TUFT1 specifically regulates Rac1 expression, in a recent study, Kawasak et al. (14) found that TUFT1 activated the mTORC1 signaling pathway by regulating the Rab GTPase, and that the interaction of TUFT1 and RabGAP1 mediated intracellular lysosome localization and vesicle transport in BC cells, while Rac1 is the substrate of mTOR. In addition, through high-throughput differential gene screening, TUFT1 was found to be associated with Rab5 and Rac1 (13). Rab5 is responsible for regulating the early stage of vesicle transport. Once activated, Rab5 recruits a number of interacting proteins, such as Rac1 and Tiam1, which play an important role in tumor metastasis (48, 49). Díaz et al. (50) found that Rab5 activation could recruit Tiam1 around the endosome, thereby leading to the activation of Rac1. Based on this, we hypothesize that TUFT1 may initiate vesicle transport through activating Rab5, thereby affecting downstream Rac1 expression. So, regulatory processes may be complex, the relationship between TUFT1 and Rac1 needs further study.

As endocrine therapy or HER2 targeted therapy is ineffective for TNBC patients. Chemotherapy is the most effective treatment at present. In addition, more than 50% of TNBCs were resistant to adjuvant chemotherapy. Because of chemotherapeutic resistance, patients often have relapse and metastasis (51, 52). In 2015, experts at St. Gallen agreed to recommend anthracyclines and taxanes as the main adjuvant chemotherapeutic drugs for TNBC. However, the use of platinum antineoplastic drugs is still controversial (53, 54). Here, we demonstrated that TUFT1 knockdown can reverse doxorubicin resistance in a TNBC xenograft tumor model. Meanwhile, TUFT1 suppression conferred sensitivity to chemotherapy and increased cell apoptosis via inhibition of Rac1/β-catenin signaling in TNBC cells. The mechanism of Rac1-mediated chemoresistance has been studied in several tumors (23, 55–57). We have found in previous studies that TUFT1 can inhibit the apoptosis of BC cells and the activation of Caspase 3 (13). Rac1 can regulate the DNA damage response, drug-induced apoptosis, and tumor metastasis by activating a number of stress-activated kinases, such as JNK and p38 kinase, which can regulate the activation of Caspase 3 (58, 59). In addition, dual specificity phosphatase-1 (DUSP1) can dephosphorylate all three family members of MAPK (ERK1/2, JNK1/2, p38 MAPK), which play a negative regulatory role in MAPK signaling pathway (60, 61). DUSP1 mediates breast cancer proliferation and chemotherapy resistance by inhibiting JNK pre-apoptotic signaling pathway (62, 63). TUFT1 can regulate DUSP1 expression in our previous studies (13), therefore, we consider whether there is a link between TUFT1/Rac1 pathway and DUSP1 to regulate downstream MAPK pathways, or whether TUFT1 directly mediates DUSP1 bypass signal to regulate apoptosis and chemoresistance of BC cells. This requires further study. CSCs as a target is a promising method for reversing chemoresistance, and activated Wnt/β-catenin pathway also can inhibit apoptosis of BC cells and confer the stemness of BC cells and lead to chemoresistance (64–66). Therefore, these results suggest that the TUFT1/Rac1/β-catenin axis can at least partially inhibit TNBC cells apoptosis and then promote doxorubicin/taxotere resistance in TNBC. Moreover, TUFT1 expression positively correlates with Rac1-GTP, and co-expression of TUFT1 and Rac1-GTP predicts poor patient prognosis in TNBC following adjuvant doxorubicin/taxotere treatment. Thus, TUFT1 may be a potential novel clinical therapy target for reversing chemoresistance in TNBC.

## Conclusions

In summary, we first systematic study on the functional mechanism of TUFT1 mediated metastasis, stemness and chemoresistance in TNBC. Our results find that TUFT1 can promotes the metastasis and stemness of TNBC cells via the RAC1/β-catenin pathway, meanwhile, TUFT1 could increase TNBC resistance to chemotherapy induced by RAC1/β-catenin pathway. Therefore, our findings suggest that TUFT1 may provide a potential target for more effective treatment of TNBC. The mechanism of TUFT1 regulating Rac1 and the mechanism of TUFT1 mediating metastatic and apoptotic bypass signaling in TNBC cells need to be further explored.

## Data Availability

The raw data supporting the conclusions of this manuscript will be made available by the authors, without undue reservation, to any qualified researcher.

## Ethics Statement

This study was carried out in accordance with the recommendations of ICMJE of guidelines, Ethics Committee of Affiliated Hospital of Hebei University of Engineering with written informed consent from all subjects. All subjects gave written informed consent in accordance with the Declaration of Helsinki. The protocol was approved by the Ethics Committee of Affiliated Hospital of Hebei University of Engineering. This study was carried out in accordance with the recommendations of International Association of Veterinary Editors guidelines, Ethics Committee of Affiliated Hospital of Hebei University of Engineering. The protocol was approved by the Ethics Committee of Affiliated Hospital of Hebei University of Engineering.

## Author Contributions

WL and BC conceived the study and provided the project direction. WL guided and performed the experiments, analyzed the data, and wrote the manuscript until the final submission version. WL, GC, LS, YZ, and JHa completed the cell experiments. WL, GC, YD, JHe, and SS assisted in performing the animal experiments.

### Conflict of Interest Statement

The authors declare that the research was conducted in the absence of any commercial or financial relationships that could be construed as a potential conflict of interest.
